# Comparison of selected prooxidant-antioxidant balance and bone metabolism indicators and BDNF levels between older women with different levels of physical activity

**DOI:** 10.1186/s12877-023-04205-5

**Published:** 2023-08-14

**Authors:** Ewa Sadowska-Krępa, Adam Rzetecki, Izabela Zając-Gawlak, Agnieszka Nawrat-Szołtysik, Michał Rozpara, Wioletta Mikuľáková, Agata Stanek, Tomasz Pałka

**Affiliations:** 1 Institute of Sport Sciences, Department of Biomedical Basis of Physical Activity, Academy of Physical Education in Katowice, Katowice, 40-065 Poland; 2 Institute of Physiotherapy and Health Sciences, Department of Physiotherapy in Internal Diseases, Academy of Physical Education in Katowice, Katowice, 40-065 Poland; 3 Institute of Physiotherapy and Health Sciences, Department of Physiotherapy, Academy of Physical Education in Katowice, Katowice, 40-065 Poland; 4 Institute of Sport Sciences, Department of Health-Promoting Physical Activity and Tourism, Academy of Physical Education in Katowice, Katowice, 40-065 Poland; 5 Faculty of Health Care, Department of Physiotherapy, University of Presov, Presov, 080 01 Slovak Republic; 6 Department and Clinic of Internal Medicine, Angiology and Physical Medicine, Faculty of Medical Sciences in Zabrze, Medical University of Silesia, Bytom, 41-902 Poland; 7 Department of Physiology and Biochemistry, Faculty of Physical Education and Sport, University of Physical Education in Krakow, Krakow, 31-571 Poland

**Keywords:** Antioxidant enzymes, Non-enzymatic antioxidants, Oxidative stress, Bone turnover, Brain-derived neurotrophic factor, Aging, Physical activity

## Abstract

**Background:**

Given a lack of studies precisely indicating how many steps elderly people should take daily for their antioxidant defence, bone metabolism, and cognitive abilities to improve, our study set out to compare the selected antioxidant, prooxidant, bone turnover, and BDNF indicators between elderly women differing in physical activity (PA) measured by the daily number of steps.

**Methods:**

The PA levels of 62 women aged 72.1 ± 5.4 years were assessed based on their daily number of steps and then were used to allocate the participants to three groups: group I (n = 18; <5,000 steps a day); group II (n = 22; from 5,000 to 9,999 steps a day); and group III (n = 22; ≥10,000 steps a day). Blood samples were collected from the participants in early morning hours and subjected to biochemical analysis for prooxidant-antioxidant balance indicators (SOD, CAT, GPx, GR, GSH, UA, MDA and TOS/TOC), bone metabolism indicators (Ca, 25-OH vitamin D, osteocalcin, CTX-I, and PTH), and BDNF levels.

**Results:**

The groups were not statistically significantly different in the activity of SOD, CAT, GPx, and GR, but their concentrations of GSH (H = 22.10, p < 0.001) and UA (H = 12.20, p = 0.002) proved to be significantly associated with the groups’ daily PA. The between-group differences in the concentrations of MDA and TOS/TOC were not significant, with both these indicators tending to take higher values in group I than in groups II and III. Significant differences between the groups were established for the concentrations of 25-OH vitamin D (H = 24.21, p < 0.001), osteocalcin (H = 7.88, p = 0.019), CTX-I (H = 12.91, p = 0.002), and BDNF (H = 14.47, p = 0.001), but not for Ca and PTH.

**Conclusions:**

Significantly higher concentrations of GSH, slightly lower oxidative stress indicators, significantly higher BDNF levels, and moderately better bone turnover indicators and resorption markers in the group taking more than 5,000 steps a day suggest that this level of PA can promote successful aging. More research is, however, needed to confirm this finding.

## Introduction

Regular physical activity (PA) is known to improve bodily functions and reduce the risk of developing circulatory diseases, type 2 diabetes, osteoporosis and even breast and large intestine cancer in people of all ages [1–3]. Even limited amounts of routine daily walking, work in the garden and household cleaning are reported to diminish the risk of falling [4] or developing Alzheimer’s disease [5]. Recent research has shown that engaging in any PA is healthier than living a sedentary lifestyle [6]. Adults are recommended to take at 7,000–8,000 steps a day [7], but Aranyavalai et al. [8] argue that older people who take more than 5,000 steps per day have a significantly lower risk of falling compared with the less active seniors. They also point out that of all types of health-benefitting PA, walking is the most convenient.

The free radicals theory of ageing holds that ageing increases oxidative stress while making the reparatory systems less efficient, which leads to greater oxidative damage and a higher risk of developing diseases and degenerative processes. However, there is also strong research evidence pointing to the ability of regular, moderate-intensity PA to bring about positive adaptive changes in antioxidant defence mechanisms [9, 10], to improve bone formation and reduce bone resorption biomarkers [11], and to stimulate cognitive function in seniors [12].

Age-related degenerative processes affect bone composition, structure, and function in in older people, making them more susceptible to conditions such as osteoporosis. Among the many mechanisms indicated by research as contributing to bone resorption [13], there are vitamin D_3_ and calcium deficiency developing with age, which may also induce secondary hyperparathyroidism [14]. The deficiency of vitamin D_3_ has been observed to occur in older populations all over the world. Lower concentration of its metabolite 25(OH)D is associated with lower absorption of calcium and reduced concentration of calcitriol, which leads to the secretion of parathormone (PTH) and, consequently, increased osteoclast activity and bone resorption that particularly affect compact bone mass [15].

While limited PA slows down osteoblast activity while increasing the expression and secretion of proteins that stimulate the activity of osteoclasts [2], mechanical forces acting on bones during physical exercise stimulate bone turnover [16]. Hence, PA is recommended as essential in the prevention and treatment of age-related weakening of the bone system [17].

Studies on neurodegenerative processes indicate that owing to its neuroprotective ability, regular, moderate-intensity PA can delay neural aging influenced by nerve cell growth factors [18]. PA has also been found to be able to improve memory and concentration and protect cognitive function in old age [19], because it stimulates the secretion of blood-transported neurotrophic factors, including the brain-derived neurotrophic factor (BDNF) [20], which is produced not only in the brain but also in working skeletal muscles. According to an increasing body of evidence, BDNF participates in many neurophysiological processes. The optimal concentration of this neurotrophin is necessary for the growth of cerebral ganglia and processes regulating synaptic and neural network plasticity. BDNF plays a vital role in learning and memorising in both young people and older adults [21]. More and more evidence links changes in the concentration of BDNF to greater vulnerability to stress and a higher risk of developing stress-related illnesses [22]. In patients suffering from neurodegenerative diseases such as Parkinson’s disease, Alzheimer’s disease, sclerosis multiplex, and Huntington’s disease, lower concentration of this protein is observed [23].

Reduced serum BDNF levels associated with lower hippocampus volume and memory problems in old adulthood [24] can be elevated by physical exercise, which is reported to improve seniors’ cognitive function in particular [25].

Given a lack of studies precisely indicating how many steps elderly people should take daily for their antioxidant defence, bone metabolism, and cognitive abilities to improve, our study set out to compare the selected antioxidant, prooxidant, bone turnover, and BDNF indicators between elderly women differing in physical activity measured by the daily number of steps.

## Methods

### Participants

The study was conducted as preliminary research. Its participants were 62 women aged 72.1 ± 5.4 years recruited from among the residents of the St. Elizabeth residential care facility in Ruda Śląska (Poland) and students at the Upper Silesian Third Age University in Chorzów who lived in their own homes. Eighty-six women volunteered to take part in the study, but 24 met the exclusion criteria and were excluded from the sample.

Exclusion criteria prevented the participation of women who had pacemakers or chronic diseases (diabetes, hyperthyroidism, oncological or metabolic diseases), used antioxidant supplements or vitamin D_3_ within one month prior to the study, or needed walking aids. Inclusion criteria required that participants were relatively healthy women, at ages ranging between 65 and 75 years old, and consenting to participate in the study.

All women underwent general and cardiac examinations to select those who could participate in the study. The enrolled women were divided into three groups differing in physical activity, which was assessed based on the daily number of steps using the concept of a graduated step index for healthy adults proposed by Tudor-Locke and Bassett [26]. Group I (n = 18) included women who took < 5,000 steps a day, Group II (n = 22) consisted of women who took from 5,000 to 9,999 steps, and Group III (n = 22) took ≥ 10,000 steps a day. The characteristics of the participants are summarised in Table 1.

**Table 1:** Basic characteristics of the studied women

Variable	Group	M (SD)
Age, years	Group I – < 5,000 steps/day	75.1 (6.4)
	Group II – 5,000–9,999 steps/day	72.1 (5.6)
	Group III – ≥10,000 steps/day	69.7 (2.8)
Body height, cm	Group I – < 5,000 steps/day	152.7 (7.4)
	Group II – 5,000–9,999 steps/day	157.1 (5.9)
	Group III – ≥10,000 steps/day	158.9 (4.4)
Body mass, kg	Group I – < 5,000 steps/day	61.0 (12.8)
	Group II – 5,000–9,999 steps/day	70.6 (10.7)
	Group III – ≥10,000 steps/day	66.5 (11.5)
BMI, kg/m^2^	Group I – < 5,000 steps/day	26.1 (5.0)
	Group II – 5,000–9,999 steps/day	28.6 (4.2)
	Group III – ≥10,000 steps/day	26.3 (4.3)

Notes: M – mean; SD – standard deviation

All women signed the consent forms and were briefed on the study protocol, which was designed following the ethical guidelines of the Declaration of Helsinki and was approved by the Bioethics Committee.

### Physical activity

The physical activity levels of the participants were measured with ActiGraph GT1M accelerometers (Manufacturing Technology Inc., FL, USA), which are provided with a step-count algorithm that minimises measurement errors associated with gait disturbances such as a “shuffling walk”. The accelerometers were worn by the women in the small pockets of elastic belts over the right iliac spine 12–16 h per day over 8 consecutive days. The first day readings were omitted from analysis to make sure that the participants’ initial reaction to the measurement procedure did not compromise data accuracy (thus, 12-hour readings from 7 days were taken for analysis). The women were also requested to record the time when measurement started (when they put on the accelerometers in the morning) and when it ended (when they removed them in the evening), as well as all activities they performed whether in groups or individually (type and duration), and all periods of inactivity (sitting, lying, being transported) longer than 10 min.

### Biochemical analysis

Fasting venous blood samples were collected for biochemical analysis by a qualified nurse in early morning hours. All biochemical tests were performed by a certified laboratory in accordance with PN-EN ISO 9001:2015 (certificate no. PW-19912-18B) and the test manufacturers’ instructions.

Part of each blood sample was immediately assayed for reduced glutathione (GSH) by a colorimetric method [27] with 5.5’-dithiobis-2-nitrobenzoic acid. The remainder was placed in the test tubes to separate plasma (BD Vacutainer PPT™ Plasma Preparation Tube, UK) and serum (BD Vacutainer™ Serum Tube, UK). Plasma was obtained by centrifuging the tubes for 10 min at 1000 × g at 4 °C (SIGMA 2-16KL, Sigma Laborzentrifugen GmbH, Germany). The obtained erythrocyte sediments were then washed three times with cold saline (4 °C). To extract serum, the test tubes were allowed to stand for 30 min for blood to clot and then were centrifuged at 1000 × g at 4 °C. Blood plasma, serum and erythrocytes were stored at − 80 °C before they were assayed.

The activity of antioxidant enzymes (superoxide dismutase (SOD, EC 1.15.1.1), glutathione peroxidase (GPx, EC 1.11.1.9), and glutathione reductase (GR, EC 1.6.4.2) was measured in erythrocyte homogenates. SOD activity was assessed using a RANSOD 125 kit (Randox, UK) and intra- and inter-assay CV of 4.11% and 6.51%, respectively. The activity of GPx was measured with a RANSEL RS505 kit (Randox, UK); the intra- and inter-assay CV for GPx were 5.83% and 4.03%, respectively. CAT and GR activity was determined by the methods proposed by Aebi [28] and Glatzle et al. [29], respectively.

Plasma malondialdehyde (MDA), a marker of lipid peroxidation, was assessed using the thiobarbituric acid (TBARS) reaction [30] by reading the absorption at λ = 532 nm shown by a multi-mode microplate reader (Synergy 2 SIAFRT, BioTek, USA). Standard curves were prepared using water solutions of 1,1,3,3-tetramethoxypropane (TMP) as standard.

The serum total oxidant status/total oxidant capacity (TOS/TOC) was measured by a photometric method (the KC5100 PerOx test kit, Immundiagnostik AG, Germany); the intra- and inter-assay CV were 2.94% and 6.74%, respectively. The concentration of serum calcium (Ca) was assayed using a colorimetric method (Cat. No. Ca590, the Randox Laboratories Ltd. diagnostic kit, UK) and intra- and inter-assay CV of 2.44% and 3.91%, respectively. The concentration of serum 25-OH vitamin D (total) was determined with the DRG 25-OH Vitamin D (total) ELISA kit (EIA-5396, DRG Instruments GmbH, Germany); the intra- and inter-assay CV were 4.4% and 8.3%, respectively.

The degradation products of C-terminal telopeptides of Type-I collagen (CTX-I) in serum were quantified using an enzyme immunological test (the Serum CrossLaps® (CTX-I) ELISA kit, Immundiagnostic systems GmbH, Germany) and intra- and inter-assay CV of 3.0% and 10.9%, respectively. The serum concentration of parathyroid hormone was measured with a PTH (Parathyroid) Intact ELISA kit (EIA-3645, DRG Instruments GmbH, Germany) and the serum concentration of osteocalcin with a Demeditec Osteocalcin human ELISA kit (DEKAP1381, Demeditec Diagnostics GmbH, Germany). The intra- and inter-assay CV for PTH and osteocalcin were 6.08% and 3.6% and 4.6% and 3.4%, respectively. BDNF levels were determined using a RayBio® Human BDNF ELISA kit (ELH-BDNF, RayBiotech, USA) and intra- and inter-assay CV < 10% and < 12%, respectively.

### Statistical analysis

The data presented below represent means (M), standard deviations (SD), medians (Me), and quartile deviations (QD). The normality of data distributions and the homogeneity of variance were determined using the Shapiro-Wilk test and Levene’s test, respectively.

Between-group differences in the level of biochemical indicators were determined by the Kruskal-Walli’s rank variance analysis (H test) and Dunn’s post-hoc tests with Bonferroni adjustment for multiple comparisons (Z test). The level of significance in all tests was set as α = 0.05. The H and Z tests were applied when non-normal data distributions prevented the use of parametric ANOVA. The effect size of physical activity on the selected biochemical indicators ($\eta _{\rm{H}}^{\rm{2}}$) was assessed, taking $\eta _{\rm{H}}^{\rm{2}}$= 0.01 - <0.06 to denote a small effect size, $\eta _{\rm{H}}^{\rm{2}}$= 0.06 - <0.14 a moderate effect size, and $\eta _{\rm{H}}^{\rm{2}}$≥ 0.14 a large effect size [31]. The statistical analysis was performed in IBM Statistics 26.0 (IBM Corporation, Armonk, NY, USA).

## Results

### Between-group differences in physical activity levels

**Fig. 1:**
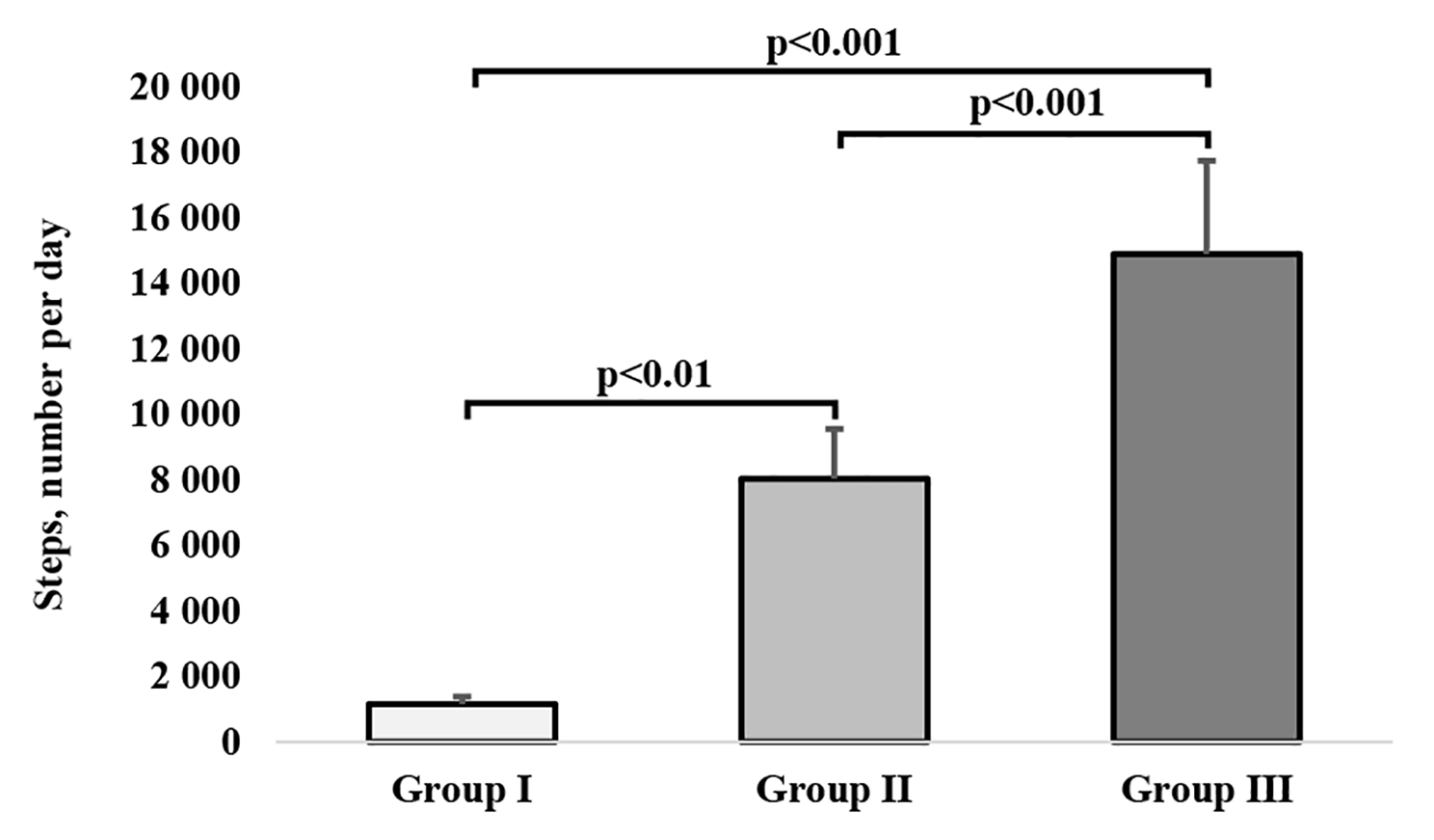
The number of steps taken per day by study groups. The bars and whiskers represent medians and quartile deviations, respectively

The H test showed that the levels of physical activity were statistically significantly different between all compared groups (H = 54.07, p < 0.001, $\eta _{\rm{H}}^{\rm{2}}$ = 0.88) (Fig. 1), and the Z test pointed to the significance of differences between groups 1 and 2 (p < 0.01), groups 1 and 3 (p < 0.001), and groups 2 and 3 (p < 0.001) (Fig. 1).

### Prooxidant-antioxidant balance indicators

**Fig. 2:**
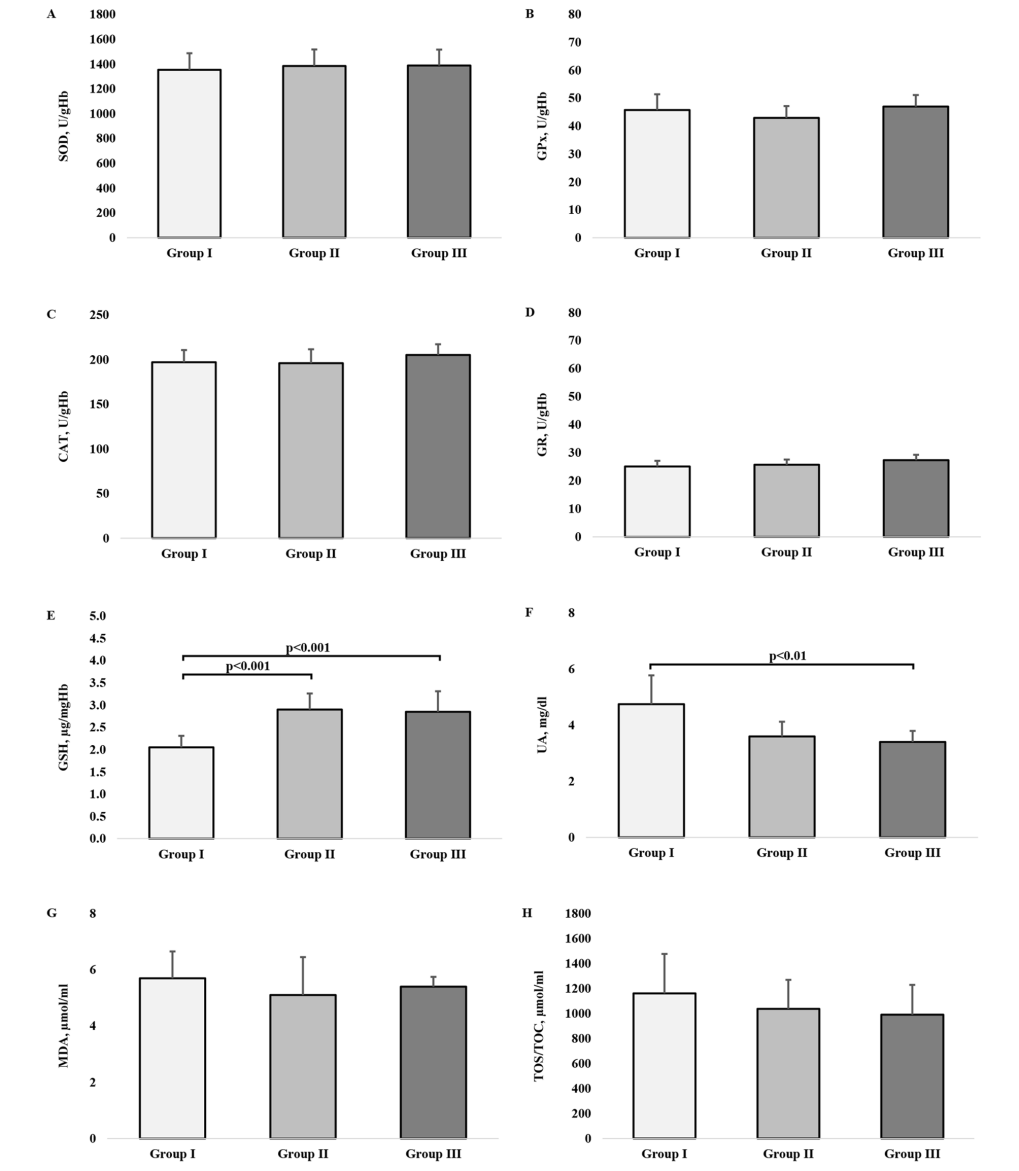
The values of prooxidant-antioxidant balance indicators by study groups. A: SOD- Superoxide dismutase, B: GPx-Glutathione peroxidase, C: CAT-Catalase, D: GR-Glutathione reductase, E: GSH-Reduced glutathione, F: UA-Uric acid, G: MDA-Malondialdehyde, H: TOC/TOC-Total Oxidative Stress. The bars and whiskers represent medians and quartile deviations, respectively

The prooxidant-antioxidant balance indicators calculated for each group by the H test are presented in Fig. 2. SOD (H = 1.86, p = 0.394, $\eta _{\rm{H}}^{\rm{2}}$ = 0.00), GPx (5.36, p = 0.069, $\eta _{\rm{H}}^{\rm{2}}$ = 0.06), CAT (H = 5.12, p = 0.077, $\eta _{\rm{H}}^{\rm{2}}$ = 0.05), and GR (H = 5.92, p = 0.052, $\eta _{\rm{H}}^{\rm{2}}$ = 0.07) activity is not related to the groups’ physical activity, in contrast with the concentration of GSH (H = 22.10, p < 0.001, $\eta _{\rm{H}}^{\rm{2}}$ = 0.34), which the results of the Dunn’s post-hoc tests show to be significantly higher in groups II and III compared with group I.

Although the concentration of UA and the level of physical activity are associated with each other (H = 12.20, p = 0.002, $\eta _{\rm{H}}^{\rm{2}}$ = 0.17), only groups I and III have significantly different levels of UA (p < 0.01).

Regarding MDA (H = 0.76, p = 0.684, $\eta _{\rm{H}}^{\rm{2}}$ = -0.02) and TOS/TOC (H = 1.48, p = 0.476, $\eta _{\rm{H}}^{\rm{2}}$ = -0.01), the between-group differences in their concentrations are not significant, but their levels show a tendency to be higher in group I compared with groups II and III (Fig. 2).

### BDNF levels

**Fig. 3:**
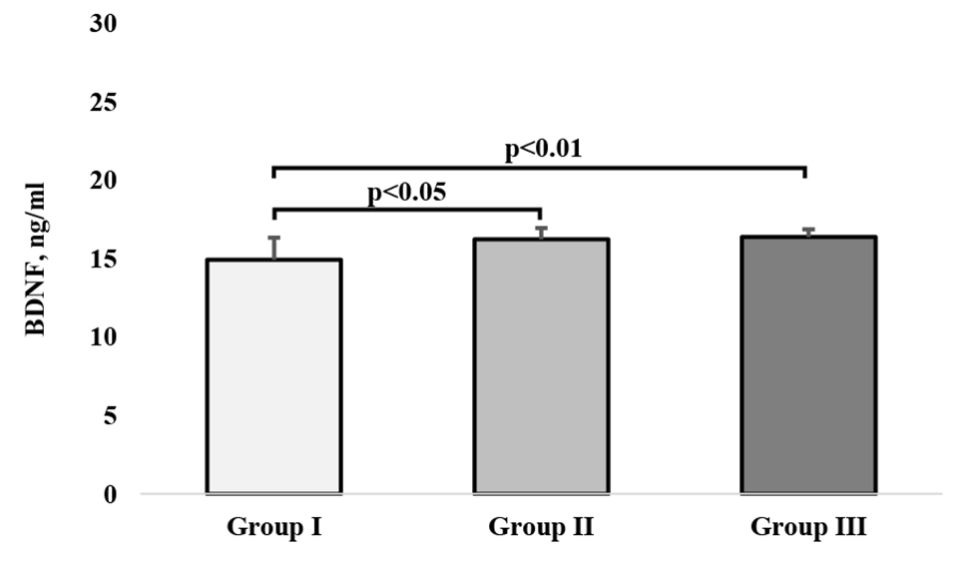
The values of BNDF (Brain-derived neurotrophic factor) indicator by study groups. The bars and whiskers represent medians and quartile deviations, respectively

The H test showed that the mean ranks for BDNF were statistically significant different (H = 14.47, p = 0.001, $\eta _{\rm{H}}^{\rm{2}}$ = 0.21), and the Dunn post-hoc pointed out that groups I and III had significantly higher values of BDNF (p < 0.05 and p < 0.01, respectively) than group I (Fig. 3).

### Bone metabolism indicators

**Fig. 4:**
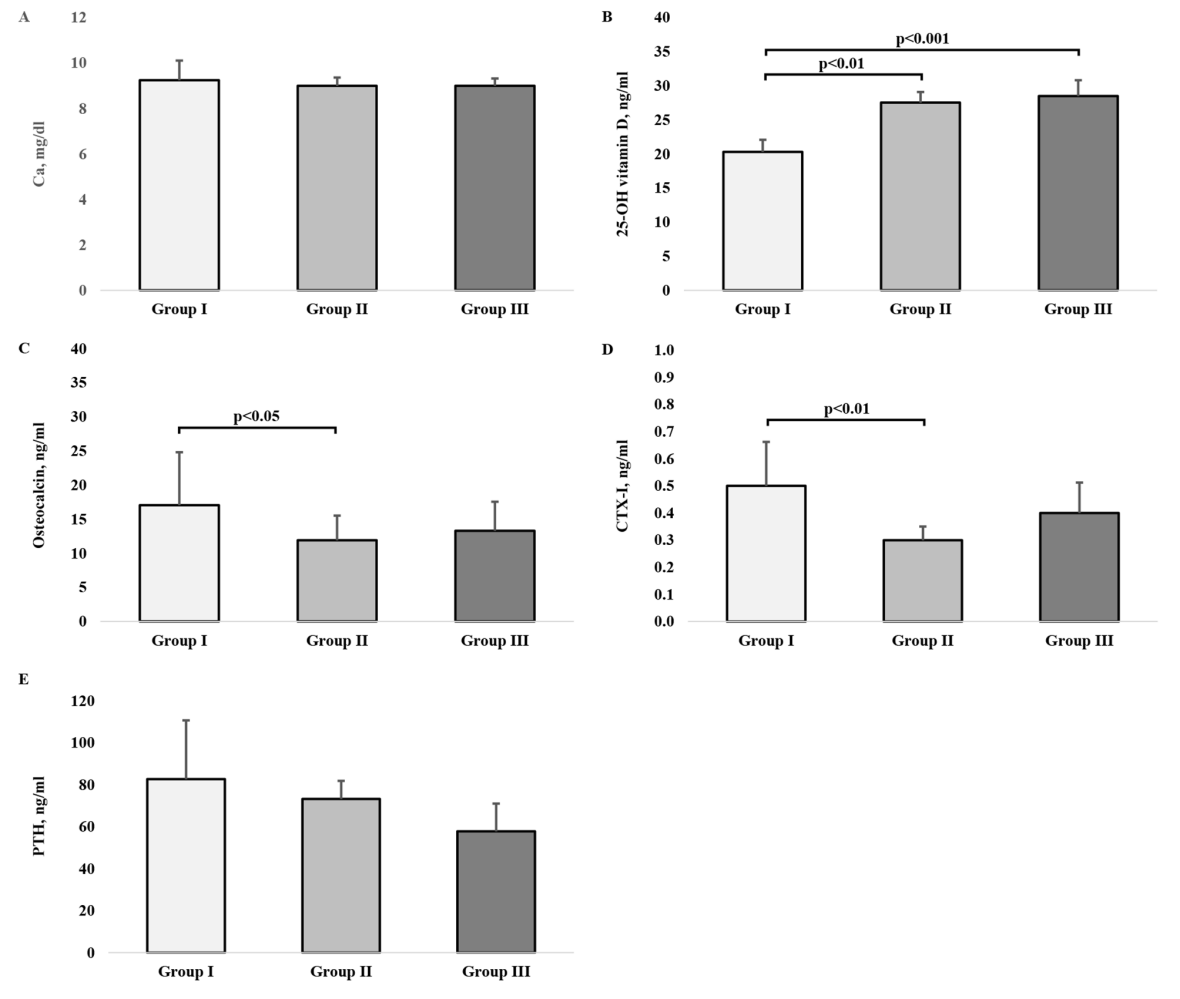
Bone metabolism indicators. A: Ca-Calcium, B: 25-OH vitamin D, D: CTX-I-C-Terminal telopeptides of type-I collagen, E: PTH-Parathyroid. The bars and whiskers represent medians and quartile deviations, respectively

The values of the bone metabolism indicators yielded by the H test show that the groups differed statistically significantly in the concentration of 25-OH vitamin D (H = 24.21, p < 0.001, $\eta _{\rm{H}}^{\rm{2}}$ = 0.38); the Dunn post-hoc tests indicated statistically significant differences in the level of this metabolite between groups I and II (p < 0.01) and groups II and III (p < 0.001). The mean ranks for osteocalcin were also significantly different between the groups (H = 7.88, p < 0.019, $\eta _{\rm{H}}^{\rm{2}}$ = 0.10); its concentration in group I was statistically significantly higher compared with group II (p < 0.05). The H test pointed out that the groups had statistically significantly different concentrations of CTX-I (H = 12.91, p = 0.002, $\eta _{\rm{H}}^{\rm{2}}$ = 0.18), and the Z tests pointed to statistically significant differences between groups I and II (p < 0.01). According to the H-test results, the concentrations of Ca (H = 4.29, p = 0.117, $\eta _{\rm{H}}^{\rm{2}}$ = 0.04) and PTH (H = 5.59, p = 0.061, $\eta _{\rm{H}}^{\rm{2}}$ = 0.06) were not statistically significantly different between the groups (Fig. 4).

## Discussion

The study sought to determine whether older women’s different levels of physical activity measured by the daily number of steps would be reflected in their antioxidant defence indicators, selected bone turnover markers, and BDNF levels.

The activity of the enzymes comprising the first line of antioxidant defence was not statistically significantly related to participants’ PA (the effect size of physical activity proved relatively small, 0.01–0.07). However, in those of them who were physically more active, particularly in the group taking more than 10,000 steps a day, the activity of the enzymes tended to be higher. The results are like the findings reported by Kozakiewicz et al. [32], who compared the activity of antioxidant enzymes between elderly persons engaging in regular, moderately intense PA and their sedentary counterparts, finding it to be higher in the former. A similar observation was earlier made by Bouzid et al. [33], who compared the activity of SOD, GPx and GR at rest and after resistant exercises between adults and older adults with different PA levels to see if regular PA stimulated the activity of the enzymes and reduced oxidative stress. Their conclusion was that PA could stimulate the activity of antioxidant enzymes despite an age-related increase in oxidative stress. Consequently, they suggested that the health benefits of exercise continue if people remain physically active.

Of all antioxidants investigated, glutathione peroxidase (GPx) proved to be the most strongly related to age and PA level. Kozakiewicz et al. [32] found that people who regularly participated in physical exercise had higher GPx levels compared with those who rarely exercised.

The second line of antioxidant defence is formed of non-enzymatic antioxidants including reduced glutathione (GSH) and uric acid (UA). GSH is a strong antioxidant because of its ability to inactivate hydrogen peroxide, organic peroxides, ROS, and exogenous and endogenous electrophilic compounds [34]. It can also chelate and inactivate metal compounds, regenerate other antioxidants, and support the repair of damaged cell components such as lipids and proteins [35]. In our study, group I had significantly lower GSH concentration compared with groups II group III; the effect size of physical-activity effect on GSH turned out to be considerable ($\eta _{\rm{H}}^{\rm{2}}$ ≥ 0.14). These results are aligned with the findings of other researchers, who found a relationship between the concentration of GSH and the level of oxidative stress and age [36].

One of the strongest non-enzymatic antioxidants is UA, which is estimated to enable as much as two-thirds of all reactions involved in the scavenging of free radicals. Notwithstanding its strong antioxidant properties, it has not yet been resolved whether an increase in its concentration is health-beneficial or health-adverse [37]. The studies on antioxidant stress in older adults suggest that a slightly elevated UA level (above its reference range of 2.4–7.0 mg/dl) may help ageing people sustain physical fitness and muscle function [38]. The UA concentration in our study was significantly associated with the level PA and tended to be higher in participants with lower PA (moderate $\eta _{\rm{H}}^{\rm{2}}=$0.06 - < 0.14).

Malondialdehyde (MDA) and total oxidative status/capacity (TOS/TOC) are markers of oxidative stress [39]. Under physiological conditions, ROS play an important role in cell homeostasis as a mediator of many physiological processes, but their increased production boosts lipid peroxidation and consequently elevates the level of MDA [40]. Although we did not find the values of the lipid peroxidation indicators to be related to participants’ PA level, the highest TOS/TOC was observed in group I (it was 14.7% greater compared with group III), which seems to indicate that a low PA level is associated with increased oxidative damage.

Vincent et al. [41] evaluated the concentration of lipid peroxidation products in older adults after 6 months of regular physical exercise. In addition to the study participants having less fat tissue and better BMI at month 6, their TOS/TOC turned out to be positively correlated with the change in muscle strength and negatively with fat tissue reduction. The findings imply that regular PA can additionally protect seniors from the adverse influence of ROS, and cardiovascular risk factors, to which people aged 65 + are particularly vulnerable.

Bouzid et al. [33], who analysed MDA concentrations in young adults and seniors at rest and after resistance exercise, did not find them to be statistically significant different between the two groups immediately after training. The following day, an increased MDA concentration was observed in all study participants. The results indicate that regular physical exercise has a positive effect on antioxidant defence, and that regular PA can improve antioxidant response and minimize damage caused by lipid peroxidation that increases with aging.

In our study, bone turnover in the participants was assessed based on the concentrations of the metabolite of vitamin D_3_, calcium (Ca), osteocalcin, CTX-I, and PTH that regulates the calcium level in the blood. Vitamin D_3_ supplementation helps prevent osteopenia and osteoporosis as the vitamin supports calcium-phosphate homeostasis, and its deficiency disturbs mineralisation processes resulting in bone mass reduction. Vitamin D_3_ also participates in cell proliferation, maturation, and differentiation, has a pleiotropic effect, and facilitates anti-inflammatory and immunomodulatory processes. The serum concentration of its metabolite ranged from 20 to 30 mg/dl in all groups, so it was suboptimal and indicated a need for supplementation [42].

Despite all three groups having the concentrations of 25-OH vitamin D in the same (suboptimal) range, significant between-group differences confirming a strong effect size of physical activity ($\eta _{\rm{H}}^{\rm{2}}$ ≥ 0.14) were noted. The highest concentration of the metabolite occurred in group 3 (28.5 ng/ml); its values in groups I and II were lower, 20.3 ng/ml and 27.6 ng/ml, respectively. A plausible explanation of why women in group III had the highest concentration of vitamin D_3_ could be their more frequent sun exposure during outdoor walks, because the skin synthesises vitamin D_3_ in response to UVB rays (> 20 mJ/cm^3^) to [43].

Vitamin D_3_ enables osteoblasts to produce osteocalcin, a protein hormone used to assess bone quality, which participates in bone mineralisation and calcium homeostasis and influences glucose concentration and fat metabolism [44]. High concentrations of osteocalcin are usually associated with faster bone metabolism and intensive bone turnover, but menopause-related osteoporosis, pathological osteoporosis, bone fractures and osteomalacia are also reported to increase its concentration [45]. In our study, the highest concentration of osteocalcin occurred in group I, indicating that the least physically active women were more at risk of developing osteoporosis than the other participants.

PTH plays a significant regulatory role in bone turnover and calcium metabolism due to its involvement in the catabolic and anabolic processes in the bones. Clinical trials have demonstrated that intravenous infusion of this hormone stimulates bone tissue formation and increase bone mass, but evidence has also been provided that increased doses of PTH contribute to bone resorption [46]. Research data indicating the ability of exercise to influence the concentration of PTH [47]. According to Bouassida et al. [46], the amount by which the concentration of PTH changes under the influence of PA depends on its duration and intensity. Other factors reported to affect the level of PTH include bone mineral content, age, sex, the level of fitness, and some hormonal and metabolic parameters (the levels of catecholamine and calcium). However, we did not find the concentration of PTH to be significantly related to the level of PA, but its highest level (82.8 ng/ml) occurred in the least physically active women (group I) and decreased with as the level of PA increased, being 73.3 ng/ml in group II and 57.9 ng/ml in group III.

The results of studies investigating the PA effect on the concentration of PTH in older adults are inconclusive. Most studies looking into the relationship between PTH and PA involved young, healthy, and trained people and usually assessed it after a single exercise session. Kristoffersson et al. [48] reported increased concentration of PTH in subjects who completed two exercise protocols involving high-intensity continuous exercises (two 21-minute bouts of running at 75% and 85% of VO_2_max, respectively) and intermittent exercises (the same exercises but separated by a 40-minute rest period). However, in the study by Maïmoun et al. [49], the PTH concentration of seven male adults did not change after 50 min of exercise below the ventilation threshold.

Regarding the calcium concentrations in our study, they were not statistically significantly different between the groups, but they, too, turned out to be related to the level of physical activity. The highest level of calcium, 9.3 mg/dl, was noted for group I; in groups III and II, it was slightly lower, 9 mg/dl on average.

The concentrations of CTX-I, the last of the bone turnover markers we investigated, differed between the study group. The highest and lowest levels of CTX-I were noted for groups I and II, respectively. Smith et al. [50] analysed a sample of studies with middle-aged and older men and women (n = 275, 212 women and 63 men, mean age 57.9 ± 1.5 years) and seniors (n = 93, 50 women and 43 men, mean age 68.2 ± 2.2 years), whose authors evaluated the effect of aerobic exercises (11 reports) and resistance training (2 reports) on participants’ bone markers. Their analysis concluded that a single aerobic exercise session increased the concentration of CTX-I in all participants and raised the level of osteocalcin in middle-aged men and the level of CTX-I in the older women. As a result, Smith et al. [50] reported that the concentrations of bone markers, including CTX-I, depended on the volume and intensity of exercise and the person’s age and sex. This finding should be tested more thoroughly by future research.

In our study we also assessed BDNF, which is essential to neural plasticity. The experiment conducted by Håkansson et al. [25], who had elderly persons perform 35-minute sessions involving physical exercises and brain-stimulating exercises to compare changes they induced in BDNF, demonstrated that even a single physical exercise session immediately increased the concentration of BDNF.

In our study, we observed statistically significant differences in the concentrations of BDNF between the groups related to their PA. The highest level of this neutrophin occurred in groups II and III, and the lowest in group I.

The effect of physical activity on BDNF depends on its duration and type, but most authors agree that moderate aerobic PA is the most effective. Unfortunately, few studies have yet attempted to assess the influence of PA on the concentration of BDNF in older people in the context of successful aging [51].

### Study limitations

The main limitations of the study include the use of a non-random sampling method to select participants and participants’ meals not being controlled in terms of vitamin D_3_ content. It was almost certainly different as some of them resided in a care facility and others in their own households.

## Conclusions

Our study demonstrated significantly greater concentrations of GSH, slightly lower oxidative stress indicators, higher levels of BDNF, and moderately improved bone turnover indicators in the participants who took more than 5,000 steps per day. The changes imply that physical activity can prevent osteoporosis and improve cognitive function in older women. They support the existing evidence that taking a minimum of 5,000 steps per day can promote successful and healthy aging, but more research is needed to confirm this finding.

## Data Availability

The datasets used and/or analysed during the current study are available from the corresponding author on reasonable request.

## Abbreviations

PA: Physical Activity
SOD: Superoxide Dismutase
CAT: Catalase
GPx: Glutathione Peroxidase
GR: Glutathione Reductase
GSH: Reduced Glutathione
UA: Uric Acid
MDA: Malondialdehyde
TOC/TOC: Total Oxidative Stress
BDNF: Brain-Derived Neurotrophic Factor
CTX-I: C-Terminal Telopeptides of Type-I Collagen
Ca: Calcium
PTH: Parathyroid
25- OH vitamin D: Total vitamin D
